# Cartilage Surgery in Overweight Patients: Clinical and MRI Results after the Autologous Matrix-Induced Chondrogenesis Procedure

**DOI:** 10.1155/2018/6363245

**Published:** 2018-05-08

**Authors:** Matthias Lahner, Christopher Ull, Marco Hagen, Christoph von Schulze Pellengahr, Kiriakos Daniilidis, Lars Victor von Engelhardt, Nina Lahner, Wolfram Teske

**Affiliations:** ^1^Joint Center Hilden, Hilden, Germany; ^2^Ruhr-University Bochum, Bochum, Germany; ^3^Department of Trauma Surgery, Berufsgenossenschaftliches Universitätsklinikum Bergmannsheil, Bochum, Germany; ^4^Biomechanics Laboratory, University of Duisburg-Essen, Essen, Germany; ^5^Agaplesion Ev. Bathildiskrankenhaus, Bad Pyrmont, Germany; ^6^Orthopädie Traumatologie Centrum (OTC), Regensburg, Germany; ^7^Faculty of Health, Witten/Herdecke University, Witten, Germany; ^8^Department of Dermatology, Venereology and Allergology, Ruhr-University Bochum, Bochum, Germany; ^9^Kath. Krankenhaus Hagen gGmbH St.-Josefs-Hospital, Hagen, Germany

## Abstract

Modern orthopaedic surgery provides a variety of techniques for cartilage repair. The Autologous Matrix-Induced Chondrogenesis (AMIC) procedure is a single-step technique with a collagen I/III scaffold for the treatment of full-thickness cartilage lesions. The aim of the study was to analyze the outcome of the AMIC procedure in overweight patients with knee cartilage defects. Overweight patients treated with AMIC surgery were followed up by clinical and MRI examination. 9 patients with a cartilage defect of the knee with a mean lesion size of 2.1 ± 1.2 cm^2^ and an average body mass index (BMI) of 29.3 were available for the follow-up. The Lysholm Score was significantly improved by the AMIC procedure (38 to 67, *p* ≤ 0.008). The VAS Score was significantly lower after the procedure (9 to 3, *p* ≤ 0.018). In the postoperative MOCART Scale, the scaffold reached defect covering of 80%. However, 2 patients had to be revised due to persisting knee pain. The AMIC procedure enhances pain reduction and gain of knee function for cartilage defects of overweight patients. However, in cases of an increased BMI, the patient had to be informed that success rate is reduced despite good defect covering.

## 1. Introduction

Orthopaedic researchers have established innovative regenerative techniques for the treatment of cartilage damage [1, 2]. Many surgical techniques were developed for coating focal cartilage joint lesions.

Current available surgical cartilage treatments include lavage and debridement, subchondral penetration procedure (microfracture technique, drilling, or abrasion chondroplasty), osteochondral autologous transplantation (OAT), and autologous chondrocyte implantation (ACI) with or without using a scaffold [3–6]. The most used technique for a single-step procedure is the microfracture (Mfx), in which a penetration of the subchondral bone layer is performed with subsequent effluent of progenitor cells from the bone marrow into the articular cartilage lesion [3–6]. Scaffold-assisted single-step techniques were developed to enhance cartilage tissue regeneration by the combination of the Mfx with biomaterial [6]. Different matrices are currently available for cartilage surgery like scaffolds with porcine collagen I/III membrane [6–8]. The collagen membrane may improve the mechanical stability and durability for endogenous cells and may provide a proper stimulus for cartilage regeneration [8]. The Autologous Matrix-Induced Chondrogenesis (AMIC) procedure is a one-step technique with the advantage of harvesting no cartilage cells, which may lead to donor-site morbidity [8].

So far, there are no studies about the outcome of scaffold-assisted techniques of overweight patients. The aim of our study was to evaluate the AMIC procedure for the treatment of focal cartilage defects of the knee joint of overweight patients.

## 2. Materials and Methods

### 2.1. Patient Recruitment

During March 2009 and February 2010, all patients presenting with nontraumatic knee pain, with a clinical unilateral symptomatic chondral lesions grades III-IV to Outerbridge [9] after failed conservative treatment of at least 6 months and a body mass index (BMI) > 25, were considered to be study participant. All patients signed informed consent to follow clinical examination and magnetic resonance imaging (MRI). Our study follows the principles of the Declaration of Helsinki. We excluded patients incapable of providing informed consent due and those with local or systemic infection, corticosteroid injection within the last 3 months, rheumatological disorders, cancer, immunosuppression, or osteoarthritis (OA) with collateral ligament instability > grade II. Clinical very common mid-size lesions between 2 and 3 cm^2^ were investigated. Higher deformity of the leg axis was excluded by preoperative whole-leg radiography.

### 2.2. Operative Technique

**Figure 1:**
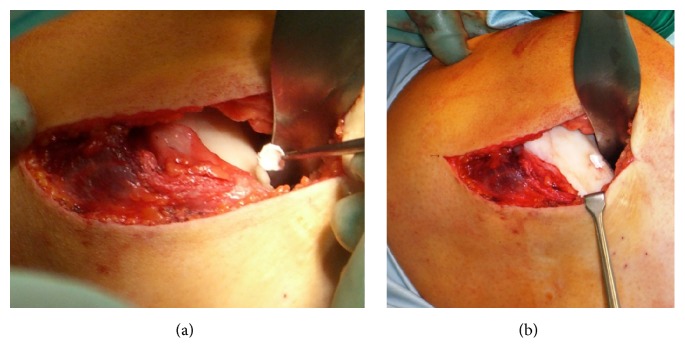
(a) The membrane was placed with the porous layer facing the bone surface. The matrix is brought into the defect. (b) The matrix membrane is glued into the defect.

First, a knee arthroscopy was performed to evaluate the extent of the cartilage lesion according to an internal standard operating procedure (SOP). Surgery was performed with the patient placed supine on an arthroscopy table. Meniscal lesions were resected during the arthroscopy. After removing the unstable cartilage, a mini-arthrotomy was performed. The chondral defect was debrided and the subchondral bone was exposed to get a sharp-edged transition to the surrounding healthy cartilage. Perforations into the subchondral bone were made with a curved awl. The cartilage defect was covered with a collagen I/III matrix of porcine origin (Geistlich Pharma AG, Wolhusen, Switzerland) to host and hold the superclot generated by Mfx. For the fixation of the matrix, a fibrin glue (Baxter Immuno, Heidelberg, Germany) was used.  Figure 1 illustrates the fixation procedure. After a fixation time of at least 5 minutes, a final inspection and wound closure were performed.

After surgery, the weight-bearing was limited to 20 kg for 6 weeks. The patients were allowed to practice their everyday activities except sports after reaching pain-free full weight-bearing.

### 2.3. Outcome Measures

Outcome measures were chosen to represent three patient-related aspects of cartilage surgery: pain at rest or under activity, knee function, and quality of life. The evaluation was performed 1 year after cartilage surgery. Pain was measured using a 100 mm visual analog scale (VAS) [10]. The preoperative and postoperative functions were assessed using the Lysholm Score (Lysholm Knee Scoring Scale) [11]. The quality of life was evaluated using the national version of the Short Form-36 (SF-36; RAND corporation) [12] as a universal, patient-reported health survey consisting of eight weighted subscales respecting physical and mental health aspects. All used outcome measures were well established and validated for patients with cartilage defects by the International Cartilage Repair Society (ICRS) [13]. Additionally, patients were asked to state their personal satisfaction. Possible answers were not satisfied, possibly satisfied, and lightly satisfied.

### 2.4. Radiological Evaluation

At least 1 year after the cartilage surgery, a 1.5 Tesla MRI was performed for evaluating resurfacing of the cartilage defect. For the evaluation, the MOCART (Magnetic Resonance Observation of Cartilage Repair Tissue) scoring system was applied [14].

### 2.5. Statistical Analysis

Student's* t*-test was used for testing the coherent data of ordinal scaled variables. The related and nonrelated samples were evaluated with the Wilcoxon test. Statistical differences were discussed to be significant at *p* < 0.05. All observations were calculated between scorings at the follow-up period examination against the preoperative scores.

## 3. Results

**Figure 2:**
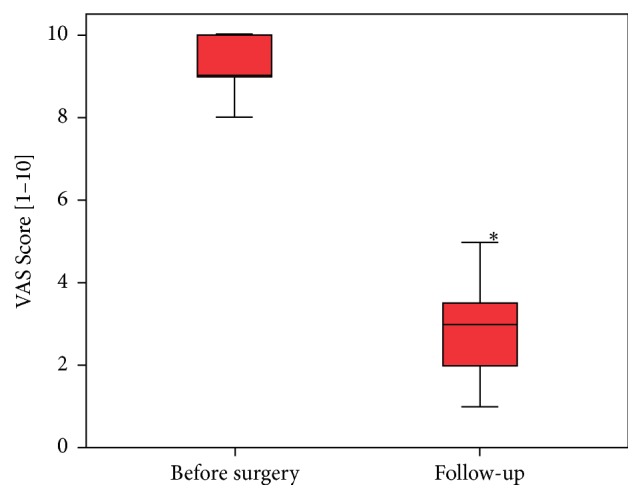
Result of the preoperative VAS Score and that at the time of the follow-up examination. The* asterisk* indicates the statistical significance.

Compared to the preoperative VAS level, patients stated a significant improvement of knee pain to a postoperative VAS score from 9 to 3 (*p* ≤ 0.018, Figure 2).

**Figure 3:**
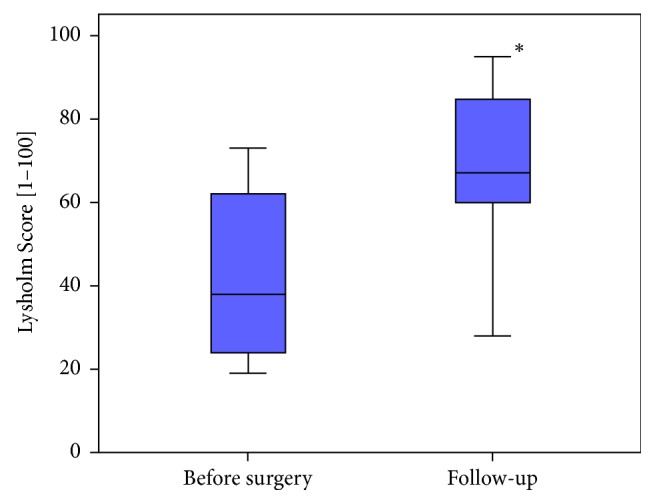
Result of the preoperative und postoperative Lysholm Score. The* asterisk* indicates the statistical significance.

The knee function that was appraised with the Lysholm Score also showed significant improvement. We achieved a postoperative score of 67 outgoing from a preoperative score of 38 (*p* ≤ 0.008, Figure 3).  Table 1 gives a breakdown of the preoperative and postoperative results.

Patients' self-assessment using SF-36 was analyzed with regard to physical and mental health. Seven of nine patients reported that the health status is better than one year ago. Therefore, in terms of the physical scale, the total value of our study group (41 ± 12.1) was lower in comparison to average value of the German population (50.2 ± 10.2).

The majority of the patients (*n* = 8) treated with AMIC procedure (*n* = 10) were examined by MRI after 14.7 months. The MOCART scoring system was applied, including seven variables to describe the morphology and signal intensity of the repair tissue. Our mean lesion size was 2.1 ± 1.2 cm^2^, representing mid-sized lesions.

**Figure 4:**
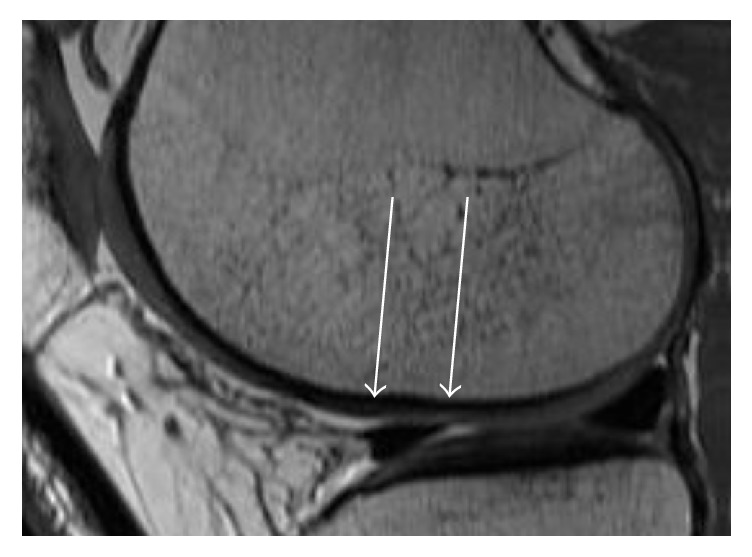
Example of MRI with sagittal orientation (1.5 T) 14 months after the AMIC procedure. In the proton density weighted sequence, the defect filling is almost complete (see with arrows) with a good integration of the reparative tissue.

There was no hypertrophy of cartilage replacement tissue in any defect (Figure 4). The majority of patients (*n* = 8) showed a defect filling more than 50% with an intact surface of the AMIC plastic. In six cases, the cartilage restoration tissue was completely integrated in the surrounding tissue. Six patients showed an isointense signal intensity of the cartilage replacement tissue with a homogeneous structure to the surrounding tissue.

The subchondral structure was intact in seven cases. No postoperative adhesions (*n* = 0) were observed. There were two revision operations due to persisting meniscal symptoms with partial medial meniscal resection and one patient received an OAT procedure due to persisting bone edema 10 months after AMIC. The results of the MRI evaluation are shown in Table 2.

The follow-up period was between 12 and 19 months with a mean of 14.7 months.

Seven out of nine patients (77.7%) questioned were subjectively highly satisfied with the results after surgery and stated that they would undergo the same procedure again if they were in the same situation as at that time. The demographic data is shown in Table 3.

## 4. Discussion

The hypothesis that was tested in this study was that the AMIC procedure results in a sustained benefit of overweight patients with focal cartilage defects. This was confirmed by our findings.

The AMIC procedure can be applied in a one-step surgery. In contrast to the ACI, there is no need to harvest healthy cartilage for application in a two-step procedure. Thus, the patient is released from a second operation with the donor-site morbidity of the harvested cartilage. So we have no situation of inflammation caused by harvest-related cartilage lesions compared to the ACI [15, 16]. Hence, the rehabilitation phase is significantly shortened with the AMIC procedure compared to the ACI.

Overweight is a well-known risk factor for osteoarthritis of the knee [17]. The group of Wluka et al. showed a correlation between primary articular cartilage defects and secondary knee osteoarthritis for a large group of patients in a four-year survey [18]. Because of these facts, our results may be helpful for the development of strategies for this growing group of patients.

This study has a few characteristics. The limiting factor of our investigation is the small number of participants. Moreover, we had no control group for ethical reason.

A further limitation of our study is the relatively short mean follow-up. So the mid-term and long-term outcomes remain to be seen, especially regarding the survey of the AMIC membrane and the overweight.

However, our study is the first survey in the literature which evaluated the outcome of matrix based cartilage surgery in overweight patients.

Our results go along with the recent publications about this resurfacing technique and can accordingly provide ideas for cases of defect situations of the knee joint [7, 19–22].

Overall, our results are in agreement with the results of Volz et al. reporting a significant improvement in clinical outcomes in patients treated with AMIC [22]. Gille showed that the results with AMIC are better in comparison to the Mfx. Moreover, numerous authors reported a progressive decrease of the Mfx technique after two years, particularly when the lesion size was larger than 2 cm^2^ [23–25]. The principles of the AMIC technique for promoting repair tissue formation were proven in an animal model [26]. However, a recent study showed that AMIC and Mfx resulted in subchondral bone cyst formation in a sheep model after 26 weeks [27]. Beck et al. showed that 11 of 12 specimens of the AMIC group leaded to an increased trabecular thickness and a decreased bone trabecular separation. However, no clinical study reported about this phenomenon.

It is critical to see that Beck et al. apply large awls to perform Mfx. Small-diameter microfracture with awls with diameter of 1.0 mm should be used for avoidance of bone cysts after Mfx [28].

## 5. Conclusions

As a conclusion, AMIC is an effective cartilage repair procedure in the knee, resulting in stable clinical results even in obese patients. Our first results should encourage orthopaedic surgeons to apply the AMIC technique in overweight patients as well, even if further investigations are required.

## Figures and Tables

**Table 1:** Comparison of the preoperative and postoperative scores.

Variable	Preoperative score	Postoperative score	*P* value
VAS Score	9 ± 2.1	3 ± 2.6	0.018^**∗**^
Lysholm Score	38 ± 21.2	67 ± 21.9	0.008^**∗**^

^*∗*^Significant at *P* < 0.05.

**Table 2:** MRI evaluation.

Variable	<40 years	>40 years	Total
	(*n* = 4)	(*n* = 6)	(*n* = 10)
Defect filling			
Complete	3	0	3
Hypertrophy	0	0	0
Incomplete	0	0	0
>50%	1	4	5
<50%	0	2	2
Integration			
Complete	4	2	6
Incomplete	0	0	0
Marginal gap	0	3	3
Gap up to 50%	0	1	1
Surface			
Smooth	4	4	8
Partially uneven	0	1	1
Largely uneven	0	1	1
Structure of the reparative tissue			
Homogeneous	3	1	4
Inhomogeneous	1	5	6
Signal intensity of defect cover			
Isointense	3	3	6
Moderately	1	3	4
Hyperintense	0	0	0
Strongly	0	0	0
Hyperintense	0	0	0
Bone marrow			
Intact	3	5	8
Not intact	1	1	2

**Table 3:** Demographic information.

Group (9)	Age (y)	Cartilage	Body Mass
		lesions	index
<40 years (3)	33.5 ± 7.8	4	25.4 ± 4
>40 years (6)	50.2 ± 5.8	7	31.3 ± 4.8

Total	44.7 ± 10.3	11	29.3 ± 5.2
